# Automated Recommendation of Research Keywords from PubMed That Suggest the Molecular Mechanism Associated with Biomarker Metabolites

**DOI:** 10.3390/metabo12020133

**Published:** 2022-02-01

**Authors:** Shinji Kanazawa, Satoshi Shimizu, Shigeki Kajihara, Norio Mukai, Junko Iida, Fumio Matsuda

**Affiliations:** 1 Shimadzu Corporation, Kyoto 604-8511, Japan; s-k@shimadzu.co.jp (S.K.); shmz@shimadzu.co.jp (S.S.); kajihara@shimadzu.co.jp (S.K.); nmukai@shimadzu.co.jp (N.M.); ji@shimadzu.co.jp (J.I.); 2 Osaka University Shimadzu Omics Innovation Research Laboratories, Osaka University, Osaka 565-0871, Japan; 3 Graduate School of Information Science and Technology, Osaka University, Osaka 565-0871, Japan; 4 Institute for Open and Transdisciplinary Research Initiatives, Osaka University, Osaka 565-0871, Japan

**Keywords:** association analysis, biomarker discovery, keyword recommendation, Medical Subject Headings terms, MeSH co-occurrence

## Abstract

Metabolomics can help identify candidate biomarker metabolites whose levels are altered in response to disease development or drug administration. However, assessment of the underlying molecular mechanism is challenging considering it depends on the researcher’s knowledge. This study reports a novel method for the automated recommendation of keywords known in the literature that may be overlooked by researchers. The proposed method aided in the identification of Medical Subject Headings (MeSH) terms in PubMed using MeSH co-occurrence data. The intended users are biocurators who have identified specific biomarker metabolites from a metabolomics study and would like to identify literature-reported molecular mechanisms that are associated with both the metabolite and their research area of interest. The proposed method finds MeSH terms that co-occur with a MeSH term of the candidate biomarker metabolite as well as a MeSH term of a researcher’s known keyword, such as the name of a disease. The connectivity score *S* was determined using association analysis. Pilot analyses demonstrated that, while the biological significance of the obtained MeSH terms could not be guaranteed, the developed method can be useful for finding keywords to further investigate molecular mechanisms in association with candidate biomarker molecules.

## 1. Introduction

**Figure 1:**
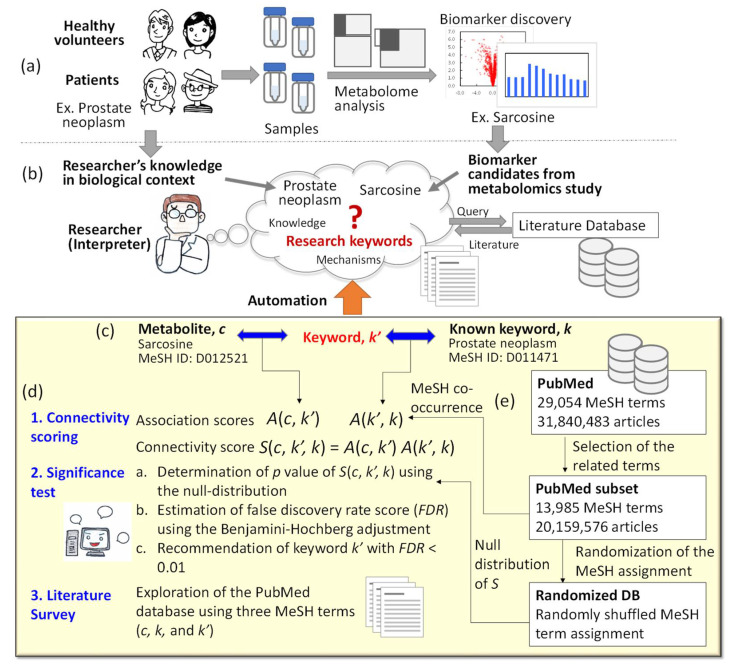
An automated method for finding Medical Subject Heading (MeSH) terms highlighting an association between metabolome data and the researcher’s knowledge. (**a**) A typical metabolomics research for biomarker discovery. (**b**) Tasks of a researcher to find research keywords suggesting a molecular mechanism. (**c**) Relationships among MeSH terms of a metabolite *c* obtained via metabolome analysis, a keywords *k*′, and the researcher’s known keyword *k*. (**d**) Novel method for keyword recommendation. The connectivity score *S*(*c*, *k*′, *k*) is determined based on the association scores *A*(*c*, *k*′) and *A*(*k*′, *k*) using the MeSH co-occurrence data derived from the PubMed subset. Significance of the connectivity score is statistically tested using null distribution of *S* derived from randomized database (DB) and false discovery rate (FDR) estimation by performing the Benjamini–Hochberg adjustment. MeSH terms below the threshold are retrieved and used to guide a literature search. (**e**) Relationship between PubMed, PubMed subset, and randomized DB used in this study.

Candidate metabolites that can be used as biomarkers for assessing disease development or drug administration have been discovered by metabolomics studies (Figure 1) [1,2,3]. To be used as a reliable biomarker, the molecular mechanism underlying the metabolic response has to be confirmed experimentally (Figure 1a) [4,5,6]. The assessment of a possible molecular mechanism is currently a bottleneck in biomarker development because it depends on the researcher’s knowledge of the metabolite’s metabolism (Figure 1b). In many cases, there is no obvious relationship between the metabolite (i.e., sarcosine) and the researcher’s knowledge of a disease (i.e., prostate neoplasm). Consequently, time-consuming mining of literature databases has been conducted to find a molecular mechanism that associates with both the metabolite and the disease (Figure 1b). The automation of this task is helpful for comprehensive exploration of molecular mechanisms that have been reported in the literature but were overlooked by the researcher.

The usage scenario or the use-case of the automated tool is as following: The intended users of the automated tool are researchers who have identified specific biomarker metabolites from a metabolomics study and who would like to identify the literature-reported molecular mechanisms that are associated with both the metabolite and their research area of interest (Figure 1b). The intended users can be biocurators who are doing biomarker discovery or fundamental biology research. They have some research keywords, such as the name of the disease, in addition to the list of metabolites. However, in most cases, intended users have limited knowledge of associations with their keywords. The first thing they do is a keyword search of the PubMed database to survey literature-reported knowledge that is overlooked by the intended users (Figure 1b). However, the intended users have had to expend a considerable amount of time and effort to check and summarize their search results because a keyword search produces only a list of research and review articles. Thus, what may help the researchers is finding useful keywords such as names of enzymes or signal transduction pathways that can explain a mechanism responsible for metabolite accumulation, and articles reporting their relationship with the disease. Moreover, these keywords should be frequently found in the literature. This suggests that intended users need an automated tool to generate a list of frequently associated and useful keywords that suggest molecular mechanisms. In the use case, the automated tool receives the user input including a metabolite name and a research keyword, and responds to it by generating a list of frequently associated and useful keywords in the PubMed database. It should be noted that further identification of true novel mechanisms that have never been reported in the literature is beyond the scope of this study.

Automation can be achieved by searching for a keyword *k*′, which has a statistically significant association with both metabolite *c* and the researcher’s known keyword *k* (Figure 1c). For this purpose, a connectivity score *S* can be determined by the methodology of association analysis, which is a collaborative filtering method for data mining [7]. Moreover, the Medical Subject Heading (MeSH) terms provide a controlled keyword vocabulary, which is a thesaurus used for indexing articles in PubMed (http://www.nlm.nih.gov/mesh/meshhome.html, accessed on 28 January 2022). There are 29,054 MeSH terms with unique IDs, such as “Sarcosine” (D012521), “Prostate Neoplasm” (D011471), among others. The intended users can find corresponding MeSH terms of metabolites and keywords using the search engine function in the “MeSH browser” webpage (https://meshb-prev.nlm.nih.gov/search, accessed on 28 January 2022). Each article in PubMed is indexed with an average of 10–20 MeSH terms. Using the MeSH indexing data, degree of association between two MeSH terms can be evaluated based on the co-occurrence frequency in each PubMed article.

MeSH terms have been used to develop informatic methods (i.e., semantic similarity, MeSH-Gram [8,9]), to visualize research trends (i.e., hierarchical structure, MeSH Sim [10]), and to estimate relationships among terms (i.e., establishment of disease-related MeSH terms [11]). Moreover, methods have been reported for suggesting keyword-based topics for unseen biomedical research articles from PubMed [12] and for annotation of scientific data with keywords from a controlled vocabulary [13]. However, an automated method to find MeSH terms associated with a biological molecule and the existing knowledge remains to be explored.

In the present study, we developed an automated method for finding a MeSH term *k*′ that associates with both MeSH terms of *c* and *k* using MeSH co-occurrence data from the PubMed database (Figure 1d,e). A method to determine the connectivity score *S* and its statistical significance was optimized using the example metabolite biomarkers for prostate cancer and type 2 diabetes. Using the developed method, we also investigated the possible connection between various metabolomes and diseases. A software package including Python scripts and MeSH co-occurrence data are available at http://www-symbio.ist.osaka-u.ac.jp/software.html (accessed on 28 January 2022).

## 2. Results

### 2.1. Preparation of Example MeSH Terms

For method development, two examples were prepared. Each example consists of three types of MeSH terms including biomarker metabolites, researcher’s known keywords, and answer keywords. Sarcosine (*N*-methyl glycine) is a non-protein amino acid that is considered a specific marker of prostate cancer [14]. A previous study has suggested that dysfunctional glycine-*N*-methyltransferase activity and transfer of a methyl group from *S*-adenosylmethionine are responsible for accumulation of sarcosine in the prostate cancer tissues and patient’s blood [15]. Using this knowledge as an example or a positive control, we investigated whether “Glycine-*N*-methyltransferase” (D050938) and “One-carbon group transferases” (D019875) could be obtained when “Sarcosine” (D012521) and “Prostate neoplasm” (D011471) were used as queries. Furthermore, we prepared a negative control in which “colorectal neoplasms” and “pancreatic neoplasms” replaced “prostate neoplasm” because they have a poor relationship with sarcosine.

Previous biomarker studies of type 2 diabetes have reported that the level of branched-chain amino acids, such as leucine, increased in patients’ blood. Branched-chain amino acids specifically act on the mammalian target of rapamycin (mTOR) receptor to initiate insulin tolerance [1,16]. This relationship was also used as an example, including the metabolite “Leucine” (D007930), the researcher’s known keyword “Diabetes Mellitus, Type 2” (D003924), and the answer keywords “Insulin resistance” (D007333) and “Mechanistic Target of rapamycin complex 1” (D000076222). MeSH term “Colorectal neoplasms” was used as a replacement of “Diabetes Mellitus, Type 2” in the negative control.

### 2.2. Development of the MeSH Term Search Method

As shown in Figure 1d, a connectivity score *S*(*c*, *k*′, *k*) among the MeSH terms of metabolites *c*, answer keywords *k*′, and the researcher’s known keyword *k*, was calculated as a product of two association scores, *A*(*c*, *k*′) and *A*(*k*′, *k*). MeSH co-occurrence data derived from the PubMed database were used to determine *A*(*c*, *k*′) and *A*(*k*′, *k*). Since the PubMed database is markedly large for method development and includes non-metabolism-related articles, a subset of PubMed was established in this study by selecting 13,985 metabolism-related MeSH terms (Data S1) and their assigned 20,159,576 articles (Figure 1e). All co-occurrence data of MeSH terms in the study were derived from this PubMed subset.

Moreover, four methods to determine association scores, *A*(*c*, *k*′) and *A*(*k*′, *k*), including cosine, Simpson, confidence, and lift, were tested in this study because they are often used in association analysis [17]. Owing to the directivity of confidence (R, from left to right; L, from right to left), the best method for determining *S*(*c*, *k*′, *k*) was selected from seven calculation methods including cosine, Simpson, lift, confidence (LR), confidence (LL), confidence (RR), and confidence (RL).

In this study, a rational threshold was set by determining the false discovery rate (FDR) from *S*(*c*, *k*′, *k*). FDR can be estimated using *p*-value of *S*(*c*, *k*′, *k*) in the null hypothesis by performing the Benjamini–Hochberg method [18]. Accordingly, a null distribution of *S*(*c*, *k*′, *k*) was established by developing randomized databases (DBs) of the subset of PubMed (Figure 1e). Randomized PubMed DBs were created by random shuffling of MeSH term assignments among the articles (Figure S1). A null distribution including 1.0 × 10^8^ *S*(*c*, *k*′, *k*) was achieved by conducting random sampling of three MeSH terms and by determining *S*(*c*, *k*′, *k*) using randomized DBs.

Using the example metabolite *c* “Sarcosine” and the known keyword *k* “Prostate neoplasm” as queries, MeSH terms *k*′ were obtained by the seven methods at FDR < 0.01 as shown in Table 1. A comparison of the search results showed that the highest number of MeSH terms (six MeSH terms) was obtained by adopting the confidence (RL) method. The list of MeSH terms, however, did not include two answer MeSH terms, “Dimethylglycine dehydrogenase” and “One-carbon group transferases” (Table S1). The second- and third-highest number of MeSH terms were obtained by the confidence (LR) (five keywords) and Simpson (four keywords) methods, respectively (Table 1). While results of the Simpson method only included one answer MeSH term, that of the confidence (LR) successfully included “Dimethylglycine dehydrogenase (ranked 3rd)” and “One-carbon group Transferases (ranked 5th)” (Table 1 and Table 2).

These scoring methods were evaluated using the negative control (Table S2). Keyword searches by the confidence (LR) method provided only one MeSH terms between “Sarcosine” and “Colorectal neoplasms”, and zero between “Sarcosine” and “Pancreatic neoplasms.” No answer keywords were included in the obtained MeSH terms (Table S2). These results demonstrate that the specific relationship among example MeSH terms can be determined using the confidence (LR) method.

The scoring methods were also evaluated using another type 2 diabetes example (Table 1). MeSH term searches using metabolite *c* “Leucine” and the known keyword *k* “Diabetes Mellitus, Type 2” as queries revealed that the confidence (LR) method provided the highest number of MeSH terms (291 MeSH terms). The obtained MeSH terms included two answers, “Insulin Resistance” (ranked 53rd) and “Mechanistic Target of Rapamycin Complex 1” (ranked 77th) (Table 1, Data S2). The second-highest number of MeSH terms was obtained by the cosine method, including one answer MeSH term, “Insulin Resistance.” The other methods found few or no MeSH terms. Moreover, the search of “Leucine” and “Colorectal neoplasms” as a negative control failed to obtain MeSH terms including answer keywords (Table S3). Based on these results, we used the confidence (LR) method to determine *S* (*c*, *k*′, *k*) throughout the study.

### 2.3. Efficient Literature Survey Guided by the Obtained MeSH Terms

A MeSH term obtained by the developed method can be a keyword that suggests a molecular mechanism between biomarker metabolites and disease. For instance, the PubMed literature search revealed that there are 76 articles assigned with the MeSH terms of both prostatic neoplasms and sarcosine (the query string is “Prostatic Neoplasms” (MeSH terms) AND “Sarcosine” (MeSH terms). The search was performed in October 2021). Instead of doing a manual survey of 76 articles, the developed method can provide MeSH terms such as “Sarcosine Dehydrogenase” as shown in Table 2. The possible roles of sarcosine dehydrogenase were investigated by a literature search using a query of three MeSH terms (the query string is “Prostatic Neoplasms” (MeSH terms) AND “Sarcosine” (MeSH terms) AND “Sarcosine Dehydrogenase” (MeSH terms)). The output of the developed software has hyperlinks to the PubMed search of the three MeSH terms (Data S2). A literature search yielded five articles. A manual survey of the five articles suggested the role of sarcosine dehydrogenase in prostate cancer. The addition of exogenous sarcosine or knockdown of sarcosine dehydrogenase could induce an invasive phenotype in benign prostate epithelial cells [14,19]. These results showed that the obtained MeSH terms can be used as a guide for performing a literature survey task in a time-efficient manner.

It should be noted that irrelevant MeSH terms were also included in the results. For instance, the MeSH term “Hepatocyte Nuclear Factor 1” is the fourth ranked keyword obtained from “Leucine” and “Diabetes Mellitus, Type 2” (Data S2). The PubMed search using “Hepatocyte Nuclear Factor 1”, “Leucine”, and “Diabetes Mellitus, Type 2” resulted in five articles about amino acid substitution, such as the effect of Ile/Leu27 polymorphism variants of the hepatocyte nuclear factor-1alpha gene on pancreatic beta-cell function in type 2 diabetes [20]. This is because the MeSH terms of amino acids have also been indexed to articles reporting amino acid substitution of proteins. Thus, caution should be maintained, especially in the case of amino acids, to avoid irrelevant keywords, due to which biomarker discovery studies often identified amino acids as biomarker candidates.

### 2.4. Summarization of the MeSH Terms by Over-Represented Analysis

The MeSH term search using metabolite *c* “Leucine” and known keyword *k* “Diabetes Mellitus, Type 2” produced a list of 291 MeSH terms (Data S2). The list was too long for researchers to investigate. To summarize the 291 MeSH terms, an over-represented analysis was performed using the tree numbers of MeSH terms [21] (Table 3; all data are available in Data S3). For instance, the MeSH term “Peptide Hydrolases” has a tree number identifier, D08.811.277.656. The tree number “D08.811.277.656” indicates that the MeSH term exists in the lower hierarchy of other MeSH terms including “Enzymes and Coenzymes” (D08), “Enzymes” (D08.811), and “Hydrolases” (D08.811.277). Moreover, there are 358 MeSH terms in the lower hierarchy of “Peptide Hydrolases”, which is 2.6% of whole 13,985 MeSH terms used in the subset database of PubMed. In contrast, among the 291 obtained MeSH terms, 28 (9.6%) were included in the lower hierarchy of “Peptide Hydrolases.” The over-representation analysis revealed that the high frequency was statistically significant (FDR = 2.05 × 10^−5^, Table 3).

The over-representation analysis of 291 MeSH terms identified 123 over-represented MeSH terms at FDR < 0.01 level (Data S3). The tree numbers of MeSH terms are also useful for narrowing down a class of MeSH terms. MeSH terms in the lower hierarchy of “Enzymes” (D08.811) seem to be relevant to a metabolism-related molecular mechanism because a metabolite directly interacts with a series of enzymes. Result of the over-representation analysis included 10 MeSH terms in the lower hierarchy of “Enzymes” (Table 3). The tree numbers showed that the 10 MeSH terms can be classified into two classes including “Peptide Hydrolases” (D08.811.277.656) and “TOR Serine-Threonine Kinases” (D08.811.913.696.620.682.700.931). The latter is a reasonable result because “TOR Serine-Threonine Kinases” is the upper hierarchy the example answer keyword, “Mechanistic Target of Rapamycin Complex 1” (D000076222). An additional literature survey also revealed that leucine is an inhibitor of a peptide hydrolase, dipeptidyl-peptidase IV (DPP IV) [22], and that DPP IV is a target of inhibitor compounds for type 2 diabetes therapy [23]. These results showed that the over-representation analysis and hierarchy of MeSH terms are useful for finding a metabolism-related molecular mechanism when a large number of MeSH terms are obtained.

### 2.5. Considerable Variations in Number of Obtained MeSH Terms among Metabolites and Keywords

Since the developed method uses MeSH co-occurrence data of the PubMed database, a search result inevitably reflects the previous research activity reported in the literature. Here, we investigated the variation in number of MeSH terms obtained by the developed method among 145 metabolites and 39 diseases.

From the targeted metabolome analysis methods [24], 145 metabolites were collected based on the availability of MeSH terms. The 39 diseases consisted of the top 20 cancers with the most frequent new cases worldwide [25] and the first 19 MeSH terms under the metabolic diseases [C18.452] in the MeSH tree hierarchy. The keyword recommendation tasks at FDR < 0.01 were executed for all 5655 combinations of metabolites and diseases (Data S4). The results showed that no MeSH terms were obtained for 4592 combinations (81%). Moreover, 1–9 and 10–99 MeSH terms were obtained for 570 (10%) and 285 (5%) combinations of metabolites and diseases, respectively. Additionally, more than 100 MeSH terms were obtained for 208 (4%) combinations. These results show that there are considerable variations in the number of obtained MeSH terms depending on the query metabolites and keywords.

**Figure 2:**
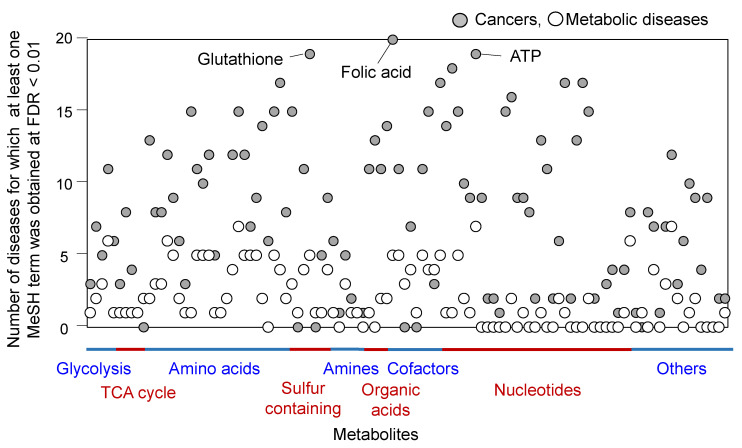
Connectivity between metabolites and diseases in literature. A pair of metabolite and disease was counted when at least one MeSH term was obtained by the developed method. The results were summarized for each metabolite against 20 cancers and 19 metabolic diseases. The complete figure with metabolite names is shown in Figure S2.

To investigate any bias in the search results, we counted pairs of metabolites and diseases when at least one MeSH term was obtained using the developed method. The results are summarized for each metabolite against 20 cancers and 19 metabolic diseases (Figure 2; all results are shown in Figure S2). The results showed that the overall connectivity of metabolites with cancers was more frequent than that with metabolic diseases. For instance, the most frequent connectivity with cancer was found for folic acid. At least one MeSH term was obtained in the combination of folic acid with 20 cancers and five metabolic diseases. The second and third most frequent connectivity were also observed for ATP (19 cancers and seven metabolic diseases) and glutathione (19 cancers and five metabolic diseases). These results indicate an intensive research activity in the cancer metabolism field. These results also coincide with the essential roles of one-carbon (folic acid), energy (ATP), and redox (glutathione) metabolism in various cancers reported in previous articles. The biases derived from the previous research activity also implied that the developed method can identify associations among MeSH terms in the PubMed literature.

## 3. Discussion

The intended users of the developed method are researchers who want to find frequently associated and useful MeSH terms from given MeSH terms of a metabolite and a known keyword (Figure 1b). In the present study, we reported a method for identification of frequent MeSH terms that have high co-occurrence frequency for both the MeSH metabolite terms and the researcher’s known keyword (Figure 1 and Table 2). The two examples demonstrated that this method could produce useful MeSH terms of responsible enzymes and signal transduction pathways such as “Sarcosine Dehydrogenase” from sarcosine and prostate neoplasm (Table 2) and “Mechanistic Target of Rapamycin Complex 1” from leucine and diabetes mellitus, type 2 (Table 3). We used simple methods in this study for proof of concept, such as MeSH terms as vocabulary, the confidence method for association scoring, *p*-value estimation using a randomized dataset, and the Benjamini–Hochberg method to control FDR (Figure 1 and Table 1). Thus, a more sophisticated recommendation can be achieved using advanced algorithms.

The use of MeSH terms as a vocabulary has several advantages and disadvantages. One of the advantages is the easy access of articles related to the MeSH terms, since they are used for indexing in PubMed (Table 2). Another advantage is the tree number of the MeSH terms [21]. While the FDR was controlled, a large number of MeSH terms were obtained depending on a pair of query metabolite and keyword (Figure 2). In such cases, the hierarchy of MeSH terms is useful for summarizing the over-representation analysis and narrowing down a class of MeSH terms. As demonstrated by the example of leucine and diabetes mellitus, type 2, a list of 291 MeSH terms was narrowed down to nine MeSH terms, as shown in Table 3.

In contrast, a disadvantage lies in the completeness of keywords [5] because the current version of the MeSH term does not include important metabolites. The incompleteness can be complemented by MeSH Supplementary Concept Record of PubMed and the CAS registry number available in the Chemical Abstracts database (https://www.cas.org/, accessed on 28 January 2022). It should be noted that the use of a larger vocabulary may increase the number of false positives or negatives [26]. Keeping this in mind, we constructed a subset of the PubMed database by selecting MeSH terms related to metabolism (Data S1), as data interpretation is currently a bottleneck in metabolomics studies. Similarly, keyword recommendations with lower false positives and negatives can be achieved by preparing suitable subsets of the PubMed database for various research purposes.

Our method depends on the co-occurrence of information in the PubMed database. Consequently, there are several intrinsic weaknesses in our method. For instance, it cannot identify true novel keywords that have never been reported in the literature in connection with the researcher’s keyword. Moreover, the method does not guarantee the production of a list of MeSH terms for any pair of query metabolites and keywords (Figure 2), as well as the biological significance of the obtained MeSH terms. The novelty or significance of MeSH terms also depends on the researcher’s knowledge. However, our method has strengths for the intended users. When no MeSH term is obtained by this method, the result is useful because the metabolite is unlikely to be a good biomarker based on a known molecular mechanism. Moreover, further literature search tasks should be stopped because no frequent MeSH terms were obtained from the PubMed-wide survey. When some useful MeSH terms are obtained and the researcher does not possess knowledge of the MeSH terms, the identified MeSH term can be considered as a research keyword for further investigation and the underlying molecular mechanism as a candidate metabolite biomarker.

## 4. Materials and Methods

### 4.1. Computational Resources and Code Availability

All recommendation functions were established using Python 3 executed in DGX-Station (CPU Intel Xeon [R] CPU E5 2698 v4 @ 2.20 GHz × 40, 20 physical cores [40 with hyper-reading], RAM 256 GB, OS Ubuntu 18.04). The Python script and MeSH co-occurrence data developed and used in this study are available at http://www-symbio.ist.osaka-u.ac.jp/software.html (accessed on 28 January 2022).

### 4.2. Acquisition of PubMed and MeSH Term Data

All literature data, including assigned MeSH term information, were obtained from the PubMed file server in June 2020 (https://ftp.ncbi.nlm.nih.gov/pubmed/baseline/, accessed on 28 January 2022). The PubMed database included 29,054 MeSH terms and 31,840,483 articles. Since the entire PubMed dataset was considerably large for research and method development purposes, we created a subset of MeSH terms related to central metabolism and biomarker research using metabolomics. The PubMed subset included 13,985 MeSH terms in categories of cells, neoplasms, nutritional and metabolic diseases, chemically induced disorders, chemicals and drugs, and phenomena and processes from 20,159,576 articles (Data S1).

### 4.3. Calculation of Connectivity Score S Using Co-Occurrence Information Derived from PubMed

We used cosine, Simpson, confidence, and lift as indicators of the association degree among the keywords [17]. When there is a set of MeSH terms $W=\left\{w_{1},\ldots ,w_{M}\right\}$ (*M* = 13,985), then a MeSH term of metabolite *c*, an answer keyword *k*′, and a researcher’s known keyword *k* are expressed as W $\left\{c,\;k,\;k^{\prime }\in W\right\}$. Moreover, in a set of articles $D=\left\{d_{1},\ldots ,d_{U}\right\}$ (*U* = 20,159,576), a subset of articles commonly assigned with MeSH terms α is expressed as $X_{\alpha }\subset D$. Thus, subsets of articles commonly assigned with *c*, *k*, and *k*′ are $X_{c}$, $X_{K\prime }$, and $X_{k}\subset D$, respectively. Here, $A(X_{c}$, $X_{k\prime })$ is used to describe the association score between *c* and *k*′ calculated using the cosine, Simpson, confidence, and lift indicators as follows:

$$\mathrm{Cosine}\;(X_{c},X_{k\prime })=\left|X_{c}\cap^{\;}X_{k\prime }\right|/\sqrt{\left|X_{c}\left|\times \right|X_{k\prime }\right|},\;$$

$$\mathrm{Simpson}\;(X_{c},X_{k\prime })=\left|X_{c}\cap^{\;}X_{k\prime }\right|/\min\left(\left|X_{c}\right|,\left|X_{k\prime }\right|\right),\;$$

(1) $$\mathrm{Confidence}\;(X_{c}\to X_{k\prime })=\left|X_{c}\cap^{\;}X_{k\prime }\right|/\left|X_{c}\right|$$

$$\mathrm{Lift}\;(X_{c},X_{k\prime })=\left|D\right|\times \left|X_{c}\cap^{\;}X_{k\prime }\right|/\left|X_{c}\right|\times \left|X_{k\prime }\right|$$

Hereafter, $A(X_{c}$, $X_{k\prime })$ is referred to as A(c,k′ ) for simplicity. The connectivity score *S*(*c*, *k*′, *k*) among the three MeSH terms, *c*, *k*′, and *k*, is determined as follows:

$$S(c,\;k\prime ,\;k)=A\left(c,\;k^{\prime }\right)\times A\left(k^{\prime },\;k\right)$$

To control directivity for confidence, we used four methods, including confidence, as follows: confidence (LR) = $A\left(k^{\prime },\;c\right)$ × $A\left(k^{\prime },\;k\right)$; confidence (LL) = $A\left(k^{\prime },\;c\right)$ × $A\left(k,\;k^{\prime }\right)$; confidence (RR) = $A\left(c,\;k^{\prime }\right)$ × $A\left(k^{\prime },\;k\right)$; and confidence (RL) = $A\left(c,\;k^{\prime }\right)$ × $A\left(k,\;k^{\prime }\right)$.

### 4.4. Construction of a Randomized DB and Estimation of FDR

A randomized DB is defined as a mock database where in the MeSH terms are randomly shuffled (Figure S1). The number of articles/keywords, the number of appearances of each keyword, and the number of keywords assigned to each article were the same as those in the actual PubMed subset DB. A random DB was constructed as follows: (1) All MeSH terms assigned to all articles in the PubMed database were removed to create a vacant database (Figure S1a); (2) MeSH terms were sorted in descending order based on the number of appearances *n* (Figure S1b); (3) each MeSH term was randomly allocated to vacant positions of *n* articles, and in cases where vacant positions were not available, the next MeSH term was allocated (Figure S1c); and (4) step (3) was repeated for all MeSH terms (Figure S1d).

MeSH terms corresponding to *c*, *k*′, and *k* were randomly sampled from W to obtain 2.0 × 10^7^ mock connectivity scores (*S*′) using a random DB. The procedure was iterated five times to generate a set of 1.0 × 10^8^ mock connectivity scores as a null distribution. For a given connectivity score *S* (*c*, *k*′, *k*) determined using the real DB, the *p*-value was determined as follows:

*p*-value = |*S*′ > *S*(*c*, *k*′, *k*)|/1.0 × 10^8^ (2)

where |*S*′ > *S*(*c*, *k*′, *k*)| indicates the number of *S*′ larger than *S*(*c*, *k*′, *k*) in the null distribution. *p*-value was corrected to false discovery rate (FDR) by performing the Benjamini–Hochberg method [18] using the ‘statsmodels.stats.multitest’ module [27].

### 4.5. Procedure for Finding MeSH Terms That Associate with Two MeSH Terms

(1) Two MeSH terms of metabolite *c* and the researcher’s known keyword *k* were prepared. The list of available 13,985 MeSH terms is shown in Data S1.
(2) For a MeSH term *k*′, the connectivity score *S*(*c*, *k*′, *k*) was determined using the confidence (LR) method and Equation (1) as follows:

$$S(c,\;k\prime ,\;k)=\mathrm{Confidence}\;(X_{k^{\prime }}\to X_{c})\times \mathrm{Confidence}\;(X_{k^{\prime }}\to X_{k})$$

(3) The *p*-value of the connectivity score *S* (*c*, *k*′, *k*) was determined using Equation (2), with a null distribution.
(4) The FDR value was obtained from the *p*-value using the Benjamini–Hochberg method [18].
(5) All MeSH terms *k*′, whose FDR levels were lower than the threshold level, were obtained as answer keywords.

### 4.6. Over-Representation Analysis

The hierarchical structure of MeSH terms (MeSH tree) was used for the over-representation analysis. For instance, the MeSH term “Glucose Metabolism Disorders” (unique ID, D044882, and tree number, C18.452.394) includes 23 MeSH terms below hierarchy; therefore, when a set of MeSH terms is known, the expected number of MeSH terms in the “Glucose Metabolism Disorders” category can be determined. The expected and actual values of the MeSH terms were used to perform an over-representation analysis using the chi-square test and the residual analysis. The FDR information of multiple tests was collected using the Benjamini–Hochberg method [18].

## Figures and Tables

**Table 1:** Comparison of the scoring methods using two example MeSH terms (false discovery rate level < 0.01).

	Example 1. Sarcosine and Prostate Neoplasm ^(1)^			Example 2. Leucine and Diabetes Mellitus, Type 2 ^(2)^		
Methods for Association Scoring	Number of Obtained MeSH Terms	Ranking of Dimethylglycine Dehydrogenase	Ranking of One-Carbon Group Transferases	Number of Obtained MeSH Terms	Ranking of Insulin Resistance	Ranking of Mechanistic Target of Rapamycin Complex 1
Simpson	4	4th	No hit	2	No hit	No hit
Lift	0	No hit	No hit	0	No hit	No hit
Cosine	1	No hit	No hit	54	No hit	No hit
Confidence (RR)	0	No hit	No hit	0	No hit	No hit
Confidence (RL)	6	No hit	No hit	4	No hit	No hit
Confidence (LR)	5	3rd	5th	291	53rd	77th
Confidence (LL)	1	No hit	No hit	0	No hit	No hit

^(1)^ MeSH terms (*k*′) were obtained from sarcosine (metabolite, *c*) and prostate neoplasm (the researcher’s known keyword, *k*). Results were checked by the occurrence of MeSH terms, “dimethylglycine dehydrogenase” and “one-carbon group transferases”. ^(2)^ MeSH terms (*k*′) were obtained from leucine (metabolite, *c*) and diabetes mellitus, type 2 (the researcher’s known keyword, *k*). Results were checked by the occurrence of MeSH terms, “insulin resistance” and “mechanistic target of rapamycin complex 1”.

**Table 2:** Medical Subject Heading (MeSH) terms (*k*′) obtained from sarcosine (metabolite, *c*) and prostate neoplasm (the researcher’s known keyword, *k*) using the confidence (LR) method at a false discovery rate (FDR) level of <0.01.

Ranking	Obtained MeSH Terms, *k*′	Co-Occurrence (*c*, *k*′) (*n*)	*A*(*c*, *k*′)	Co-Occurrence (*k*′, *k*) (*n*)	*A*(*k*, *k*′)	*p*-Value	FDR	PubMed Search Hit ^(1)^
1	Sarcosine Dehydrogenase	25	0.431	5	0.086	1.00 × 10^−8^	1.4 × 10^−4^	5
2	Sarcosine Oxidase	38	0.245	7	0.045	8.00 × 10^−8^	5.6 × 10^−4^	7
3	Dimethylglycine Dehydrogenase	15	0.326	1	0.022	1.70 × 10^−7^	7.9 × 10^−4^	1
4	Glycine N-Methyltransferase	19	0.075	14	0.055	3.00 × 10^−7^	1.0 × 10^−3^	6
5	One-Carbon Group Transferases	1	0.019	3	0.056	3.38 × 10^−6^	9.4 × 10^−3^	7

^(1)^ Based on the consideration of three MeSH terms of metabolite *c*, known keyword *k*, and answer keyword *k*′, a query term for PubMed (https://pubmed.ncbi.nlm.nih.gov/, accessed on 28 January 2022) search was created as prostate neoplasm “sarcosine” (MeSH Terms) AND “prostate neoplasm” (MeSH Terms) AND “*k*′” (MeSH terms). PubMed searches were conducted in October 2021.

**Table 3:** MeSH terms under the enzyme (D08.811) in the over-representation analysis of the 291 MeSH terms obtained from “Leucine” and “Diabetes Mellitus, Type 2” at a false discovery rate (FDR) level of <0.01 ^(1)^.

MeSH Tree ID	MeSH ID	MeSH Term	Number of Obtained MeSH Terms in the Lower Hierarchy	Total Number of MeSH Terms in the Lower Hierarchy	*p*	FDR
D08.811.277.656	D010447	Peptide Hydrolases	28	358	5.32 × 10^−6^	2.05 × 10^−5^
D08.811.277.656.350	D020689	Exopeptidases	10	35	4.44 × 10^−16^	5.53 × 10^−15^
D08.811.277.656.350.100	D000626	Aminopeptidases	2	6	5.96 × 10^−5^	0.000196
D08.811.277.656.350.350	D004152	Dipeptidyl-Peptidases and Tripeptidyl-Peptidases	2	3	1.92 × 10^−9^	1.09 × 10^−8^
D08.811.277.656.350.555	D045727	Metalloexopeptidases	3	10	4.13 × 10^−6^	1.63 × 10^−5^
D08.811.277.656.675.555	D045727	Metalloexopeptidases	3	10	4.13 × 10^−6^	1.63 × 10^−5^
D08.811.277.656.837	D043484	Proprotein Convertases	4	9	1.53 × 10^−11^	9.91 × 10^−11^
D08.811.913.696.620.682.700.931	D058570	TOR Serine-Threonine Kinases	3	5	4.08 × 10^−12^	2.80 × 10^−11^
D08.811.913.696.620.682.700.931.500	D000076222	Mechanistic Target of Rapamycin Complex 1	2	2	7.02 × 10^−14^	5.48 × 10^−13^

^(1)^ All MeSH terms in the over-representation analysis are available in Data S3.

## Data Availability

The data and Python scripts used in this study are available at http://www-symbio.ist.osaka-u.ac.jp/software.html (accessed on 28 January 2022).

## Supplementary Materials

The following are available online at https://www.mdpi.com/article/10.3390/metabo12020133/s1, Figure S1: Construction of a randomized PubMed database (DB), Figure S2: Connectivity between metabolites and diseases in literature, Table S1: Medical Subject Heading (MeSH) terms recommended based on sarcosine and prostatic neoplasms using confidence (RL) method at a false discovery rate (FDR) level of <0.01, Table S2: Comparison of the scoring methods using the two negative control example MeSH terms (false discovery rate level < 0.01), Table S3: Comparison of the scoring methods using the two negative control example MeSH terms (false discovery rate level < 0.01), Supplementary manual of software, Data S1: Subset of MeSH Terms related to primary metabolism metabolomics, Data S2: MeSH terms recommended from metabolite (Leucine (D007930)) and known keyword (diabetes mellitus, type 2 (D003924)) (FDR < 0.01), Data S3: Result of the over-represent analysis of the 291 MeSH terms recommended from leucine and diabetes mellitus, Type 2, Data S4: Numbers of MeSH terms obtained by the developed method among 145 metabolites and 39 diseases (FDR < 0.01).
